# Patient-sharing networks among Finnish primary healthcare professionals taking care of patients with mental health or substance use problems: a register study

**DOI:** 10.1136/bmjopen-2024-089111

**Published:** 2025-01-02

**Authors:** Laura Hietapakka, Timo Sinervo, Visa Väisänen, Ripsa Niemi, Mai Gutvilig, Outi Linnaranta, Jaana Suvisaari, Christian Hakulinen, Marko Elovainio

**Affiliations:** 1 Finnish Institute for Health and Welfare, Helsinki, Finland; 2 Faculty of Social Sciences, University of Helsinki, Helsinki, Finland; 3 Department of Health and Social Management, University of Eastern Finland, Kuopio, Finland; 4 Department of Psychology, University of Helsinki, Helsinki, Finland

**Keywords:** Primary Health Care, REGISTRIES, Health Services, Health Workforce

## Abstract

**ABSTRACT:**

**Objectives:**

Patient-sharing networks based on administrative data are used to understand the organisation of healthcare. We examined the patient-sharing networks between different professionals taking care of patients with mental health or substance use problems.

**Design:**

Register study based on the Register of Primary Health Care visits (Avohilmo) that covers all outpatient primary health care visits in Finland.

**Setting:**

We used the register data covering the visits for the service providers of seven municipalities, adult patients with at least one visit to a health and social service centre within one of the municipalities and visits during the year 2021.

**Participants:**

We first selected patients with mental health or substance use problems based on psychiatric diagnoses and information on service type and then identified the professionals (N=1566) visited. A patient-sharing relationship was defined between two professionals if a same patient had visited both of them at least once.

**Primary outcome measures:**

We analysed the potential associations of the network structure and the nodal attributes (municipality, belonging to a certain occupational group and the service type) with nodal formation using Exponential Random Graph Models.

**Results:**

The main findings showed that two professionals were more likely to share patient(s) when they belonged to the same occupational group, provided similar types of services or worked in the same municipality. Being a physician was associated with having more connections to other professionals than belonging to other occupational groups (OR for nurses 0.70, 95% CI 0.69 to 0.7 and for other occupations 0.83, 95% CI 0.81 to 0.84). Shared patients among different professionals were also more probable when the patients were shared with the professionals working within mental health or substance use services compared with outpatient healthcare services (OR 1.64, 95% CI 1.61 to 1.67).

**Conclusions:**

Patient-sharing contacts were mainly homogenous, supporting the tendency of people to have connections with similar people. The results also highlight the role of the physicians as important partners in the patient-sharing networks.

**Strengths and limitations of this study**

- We used a large national register that includes all the different professionals working in public and private sectors in primary care in Finland and thus a comprehensive and representative view to the patient-sharing relationships between professionals was obtained.
- The data included information only on a limited area in Finland and visits only from a 1 year time-period.
- Sharing same patients generally indicates that the professionals also work together and share work-related information with each other. However, this interpretation cannot be confirmed by using the register data.

## Introduction

 Mental health disorders, including substance use disorders, are recognised as considerable public health challenges.^1 2^ In Finland, the demand for mental health services has increased already during the 2010s and the COVID-19 pandemic further increased the number of patients seeking treatment.^3^ The demand for mental health services causes strain to primary healthcare which should be able to treat mild and moderate mental health and substance use problems and operate as the gatekeeper to specialised healthcare. National Mental Health Strategy and Programme for Suicide Prevention 2020–2030^1^ points out that Finnish mental health services have not been developed at a comparable rate to provide treatment and services for somatic illnesses.

Manifold collaboration between different professionals and services, care coordination and care continuity have been highlighted as key factors for appropriate care delivery for patients with mental illness.^4^ However, to develop these issues, better understanding of how patient care is managed across different specialists and institutions is needed.^5^ Several factors related to the patients as well as the service systems may affect the use of services. First, persons in need of mental health or substance use services often have complex conditions and thus multiple service needs.^6^ If these needs are not met in primary care services or if access to the services is difficult or not available, the patients may instead end up using acute care services, which is not an optimal situation.^7^

Second, in Finland, health services are separated under different administrative sectors and funding sources. During the data collection of this study, the Finnish health services were organised by the municipalities (local authorities) that were responsible for financing primary and secondary care services. Most of the health spending in Finland goes to outpatient care.^8^ Outpatient health services, including mental health services, are provided by health and social service centres (HSSCs) and outpatient departments of psychiatric hospitals.^9^ Other comparable service providers are the Finnish student health service and occupational healthcare. These primary care level services are the first-contact care and act as gatekeepers to secondary care. Psychiatric services in secondary care are responsible for treating the more severe mental disorders but a referral is needed to access these services.^8 10^

Third, mental health services have been integrated to primary healthcare with different intensities.^2^ In Finland, primary healthcare professionals (physicians and nurses) either guide the patient to contact the mental health professionals or consult them on their own. The role of psychiatric nurses has been increased, and psychosocial treatments and psychotherapies can be started in HSSCs.^11^ In some municipalities or organisations, the mental health professionals work under the same administration or even in the same team as other primary care professionals in HSSCs, and in other cases they might work in separate organisations but can be consulted when needed. These local variations of how the mental health services have been arranged can either enhance or hinder collaboration between professionals and organisations.

Challenges related to collaboration between professionals in healthcare reflect social processes that can be identified by social network analysis (SNA). SNA has become a markedly popular approach within the field of health research during the past decades.^12^ Social network studies in healthcare settings have used the so-called patient-sharing relationships to explore the structure of patient care in administrative data. In these studies, two professionals are considered to be connected to each other if they both deliver care to the same patient.^13^ Patient-sharing networks have been hypothesised to reflect aspects of collaboration, although sharing a patient does not automatically mean that the professionals actually cooperate. However, a previous study using both questionnaires and claims data found that physicians were more likely to report having a professional relationship with another physician if they shared patients.^14^

Most of the previous studies exploring patient-sharing networks using administrative data have focused on physician relationships, hospital context and used data from the USA.^13^ In this study, we examined the relationships of all different professionals taking care of patients with mental health and substance use issues in primary care by using a large Finnish register data. In addition to providing descriptive network information at the municipality level, our aim was to investigate whether there are general structural patterns related to nodes (healthcare professionals) that exist similarly beyond the level of a single municipality. To do this, we used exponential random graph models (ERGMs) that account for the complex dependencies within networks.^13^ More specifically, we examined, which local (municipality level network characteristics) and individual factors (occupational group and service type) influence patient-sharing network structure. Finding patterns that describe a professional’s networks can provide useful information for healthcare organisations for understanding and developing their services.

## Methods

### Data

Data were collected from the Register of Primary Healthcare visits (Avohilmo) maintained by the Finnish Institute for Health and Welfare (THL). This register covers all outpatient primary healthcare visits in Finland. We used a sample of the register that covered the visits for the service providers in seven municipalities (all located in one wellbeing services county which is responsible for organising primary and secondary healthcare as well as social care and rescue services for their residents). In terms of service needs as measured by national relative need indices, the study region represents an average region in Finland. We further limited this data to adult patients (over 18 years) that had at least one visit to an HSSC within one of the municipalities between 1 January 2021 and 31 December 2021.

In the register, each service user has a unique personal identification code included in the visit information. Using this code, we selected only those clients who had visited primary care services for reasons related to mental health or substance use. A visit was identified as being related to mental health or substance use if the information regarding the visit included: (1) a psychiatric diagnosis based on the International Classification of Diseases, 10th revision, F-codes (excluding dementia, mental retardation and specific developmental or speech disorders), (2) a psychological symptom or diagnosis based on the International Classification of Primary Care-2, P-codes (excluding specific learning problems, dementia and mental retardation) and/or (3) the service type was marked as relating to mental health or substance use. Service types in the register refer to the different types of primary care services that the professionals are instructed to use when marking the information regarding the visit. For example, mental health service code refers to the different mental health services, such as therapy or support for mental health problems, provided by mental health experts like psychiatric nurses.

We wanted to include only the visits made by the patients to healthcare or social services (including remote visits) and thus we excluded the visits where the professional visited the patient (home care visits) from the data. These excluded visits are mainly provided for older people needing help with daily activities or nursing services at home. We also removed the visits from the code ‘other services’ because it was not possible to know what kind of visits they referred to (the professionals are instructed to use that code if the other codes are not applicable). Additionally, we excluded visits to occupational healthcare because most of the professionals in those services did not have information on their occupational group.

Finally, we further excluded patients, who had only one visit to the forementioned service providers during the 1 year time-period, as well as patients, whose every visit during the year was to the same (one) professional. This was done using the unique ID codes of the professionals in the register. The rationale for these was to exclude the visits that would not add information on collaboration (ie, in cases when the client’s needs were satisfied with just one visit or with the help of only one professional, interprofessional collaboration was obviously not needed). Consequently, the professionals who did not have an ID code or information on which occupational group they belonged to were excluded from the final data.

From the register data, we included information on the seven municipalities (referred to as municipalities A–G), the occupational group of the professionals (divided into three categories: 1=physicians, 2=nurses or 3=other professionals such as psychologists and physiotherapists) and the service type of the visits (1=outpatient healthcare, referring mostly to the long-term illness follow-up visits to general practitioners (GPs) and nurses; 2=mental health and substance use and 3=other services such as student health services or physiotherapy). A small number of professionals had visits in more than one occupational group, service type or municipality (see tables1  2). For the network analyses, we allowed a professional to have visits in only one municipality, one occupational group and one service type. This was done by assigning the professional to the group (municipality, occupation and service type) in which they had the most visits in the register.

**Table 1:** Groups of different professionals and service types, number of visits and number of professionals in the data

Groups of professionals and service types	No. of visits	No. of professionals
**Professionals**		
1=physicians		
Senior physicians, ward physicians, specialists, etc	5750	55
General practitioners (GPs) and other physicians	34 765	243
Dentists	3921	113
2=nurses		
Registered nurses, etc	75 806	579
Public health nurses	12 634	237
Practical nurses and nurse’s aides	2234	71
Dental nurses, dental hygienists, etc	1008	98
3=other professionals		
Physiotherapists, occupational therapists, etc	5824	139
Psychologists, psychotherapists, etc	5265	30
Social workers, social counsellors, etc	961	15
Other professionals (eg, secretaries, leaders, pharmacists)	2052	12
**Service types**		
1=outpatient healthcare services (GP and nurse visits)		
Outpatient healthcare visits	64 916	645
Other healthcare visits (screenings, vaccinations, etc)	14 664	473
2=mental health and substance use services		
Mental health services	34 257	84
Substance use services	11 033	17
3=other services		
Maternity clinics, etc	7322	142
School and student health services	5773	166
Physiotherapy and other therapy services	6528	183
Clinical social work	843	8
Dental services	4884	188
All professionals	150 220	1592*
All service types	150 220	1906†

* Some of the professionals had visits under two different job titles.

† Some of the professionals had visits under different services.

**Table 2:** Number of patients, visits and professionals in the municipalities in the data

Municipality	No. of patients	No. of visits	No. of professionals
A	576	6294	103
B	1114	27 104	220
C	1241	13 320	180
D	570	4878	66
E	253	2017	40
F	6977	95 104	1162
G	155	1503	32
All	10886*	150 220	1803*

* Some patients and professionals had visits in more than one municipality.

The information on the number of patients, visits and professionals in the different phases of constructing the final data is presented in online supplemental file 1. The final data included 150 220 visits, 8517 patients and 1566 professionals.

### Constructing the patient-sharing network

We identified a relationship between two professionals (nodes) if a same patient had visited both of them at least once during the year 2021. Edges described how many patient-sharing connections between professionals there were in the data. The initial healthcare professional-patient network consisted of connections between patients and the healthcare professionals they visited. This kind of a network is generally known as a bipartite network, where there are two distinct sets of nodes (in this case, healthcare professionals and patients) and ties can be formed only between the two sets, not within.^13^ In other words, patients can only be connected to professionals, not other patients and vice versa. To form a unipartite (or one-node) patient-sharing network, healthcare professionals were connected if they were both tied to at least one same patient. The resulting patient-sharing network contains nodes (professionals) connected by edges (shared patients).

### Statistical analyses

To describe the network characteristics first at the municipality level, we calculated the number of edges (contacts between professionals) within each municipality, the mean degree (average number of contacts between professionals within the municipality), density (the number of contacts between professionals divided by the number of all possible contacts within the municipality), mean distance between contacts (how many professionals are needed for two professionals to connect to each other) and transitivity (the probability that if professional A has shared patient(s) with professionals B and C, then professionals B and C have also shared patient(s)) within each municipality. To illustrate our networks, we used sparse stress layouts that have shown to offer a good approximation of the target distances between nodes and is not computationally expensive for large graphs.^15^

To test whether there were significant patterns of patient-sharing relationships beyond the level of a municipality, we analysed the potential associations of the network structure and the nodal attributes (municipality, belonging to a certain occupational group and the service type) with nodal formation using ERGM.^16^ ERGMs are analogous to logistic regression; they predict the probability that a pair of nodes in a network will have a tie between them (in this case, a shared patient between healthcare professionals). One important advantage of ERGMs is that, as opposite to regression analyses, they can be used on a relational data (which patient-sharing data always are) since they do not require assumptions of independence. ERGMs are also scalable as the structure of the graph is represented by locally determined explanatory variables and the choice of these variables is quite flexible.^17^More generally, ERGMs are statistical models that test whether an observed network shows theoretically hypothesised structural tendencies.

We used two ERGM terms, called nodefactor and nodematch, to explain the structures of the patient-sharing networks. Nodefactor refers to the number of times nodes with a certain categorical nodal attribute appear within the edgeset. This was used for testing, for example, whether being a certain professional predicted having more connections than belonging to other occupational groups. Nodematch refers to the number of edges whose incident nodes match to the value of a certain nodal attribute. This is related to the so-called homophily principle, that is the tendency of people having contacts with similar people.^18^ We used nodematch to test whether the professionals had more contacts with professionals working in the same municipality, same profession or within same service type as they did. Bayesian information criterion (BIC) was used to compare how well the hierarchical models fit the data. The lower the BIC is, the better the model fit. The statistical analyses were performed using the programme R V.4.3.1.^19^ We used the packages ‘tidyverse’ for data manipulation,^20^ ‘igraph’ and ‘sna’ for network measures,^21 22^ ‘ggraph’ for network visualisations^23^ and ‘statnet’ (ergm) for ERGM.^24^,^26^

### Patient and public involvement

Patients and/or the public were not involved in this study.

## Results

Most of the professionals belonged either to the group of nurses or group of physicians (table 1). Most of the visits were registered as outpatient healthcare services (GP and nurse visits) and approximately one-third were registered as mental health or substance use services (table 1). The smallest municipality (G) in the data consisted of 155 patients and 1503 visits, compared with the largest municipality (F) with 6977 patients and 95 104 visits (table 2). The final data consisted of 1566 professionals and 145 105 edges (contacts between professionals).

The network characteristics within each municipality are shown in table 3. One of the municipalities (F) was much larger than others in terms of the number of nodes which also affected the network metrics. This municipality’s patient-sharing network had lower density and transitivity metrics than others. Other municipalities seemed to have equally high number of patient-sharing connections based on the transitivity measure. Two professionals were connected to each other (mean distance) through approximately two other professionals in all of the municipalities.

**Table 3:** Network metrics of the individual municipalities

Municipality	No. of edges	Mean degree	Density	Mean distance	Transitivity
A	2572	49.94	0.49	1.95	0.72
B	13 687	124.43	0.57	1.89	0.78
C	7067	78.52	0.44	1.97	0.73
D	1210	36.67	0.56	1.94	0.73
E	433	21.65	0.56	2.17	0.75
F	109 328	188.17	0.16	2.04	0.52
G	341	21.31	0.69	2.40	0.84

No. of edges=the number of contacts between professionals.

Mean degree=average number of contacts between professionals within the municipality.

Density=the number of contacts between professionals divided by the number of all possible contacts within the municipality (dependent on the number of nodes in the network).

Mean distance=how many professionals are needed for two professionals to connect to each other.

Transitivity=the probability that if professional A has shared patient(s) with professionals B and C, then professionals B and C have also shared patient(s) (independent of the number of nodes in the network).

**Figure 1:**
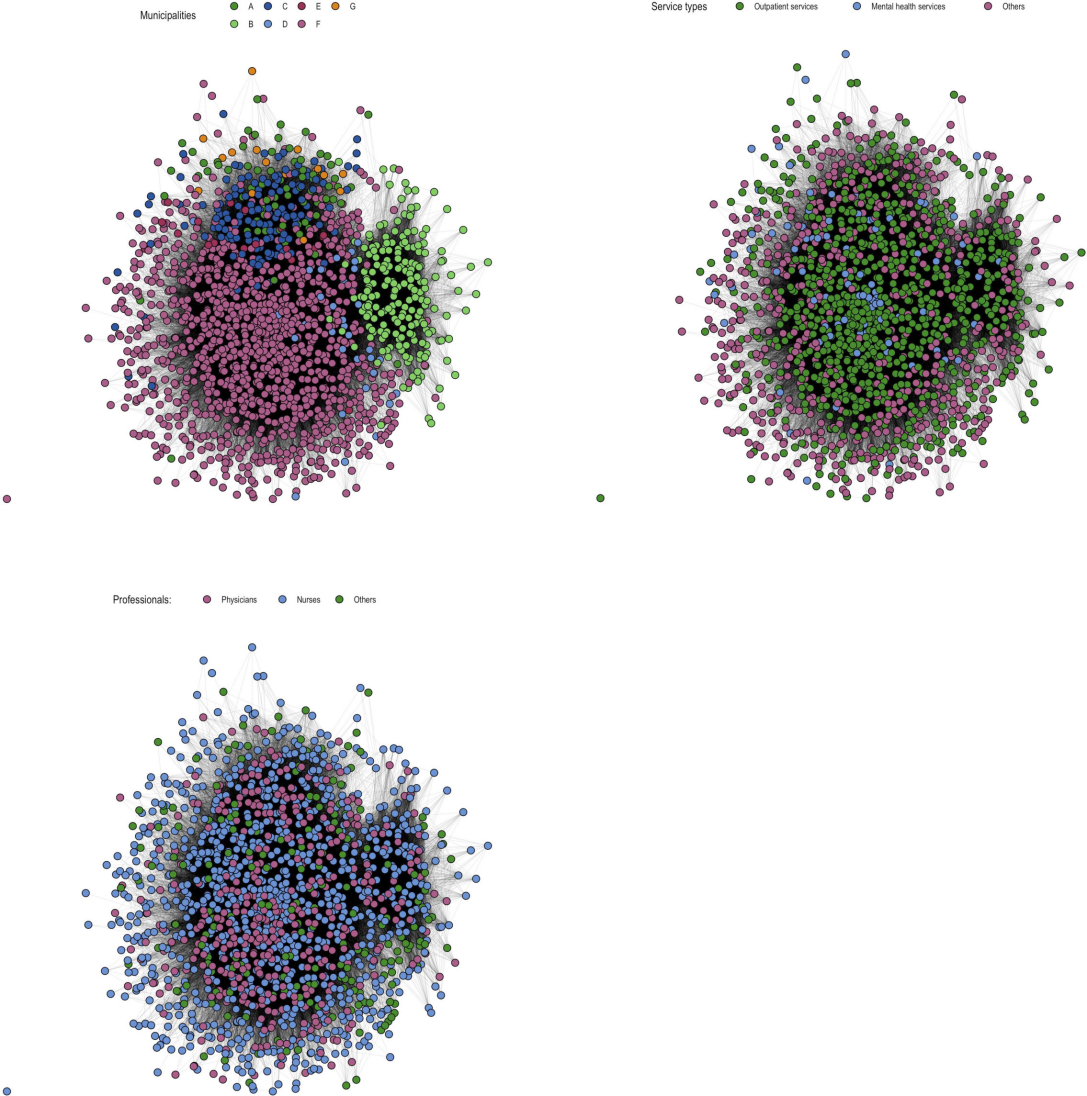
Patient-sharing network graphs within municipalities, service types and professionals

Figure 1 shows patient-sharing network graphs within municipalities, service types and professionals. The coloured nodes visually highlight the results of the ERGM, how the different nodes are clustered together based on the patient-sharing relationships in the data. The results of the ERGMs (table 4) explain the degree to which each variable explains dyadic relationships or, in other words, connections between professionals. In our null model (model 1), we estimated the simplest possible model, which only includes a term for tie density, that is, the number of connections between professionals. In model 2, we added information on municipality. Namely, we tested the main effects for contacts within municipalities (what is the professionals’ likelihood of sharing a patient depending on the municipality to which they belong) and the municipality-based homophily (is the professional more likely to share a patient with another professional from the same municipality). In model 3, we further added terms for the main effects of occupational groups and occupational group-based homophily. In model 4, we added terms for the main effects of service types and service-type homophily.

**Table 4:** ERGM analysis of municipality, occupational group and service type

	OR (CI)			
	Model 1	Model 2	Model 3	Model 4
Density (edges)	0.13 (0.13 to 0.14)*	0.02 (0.02 to 0.02)*	0.03 (0.03 to 0.03)*	0.04 (0.04 to 0.04)*
Nodefactor.municipality F (ref.)				
Nodefactor.municipality A		2.33 (2.28 to 2.39)*	2.42 (2.36 to 2.48)*	2.74 (2.67 to 2.81)*
Nodefactor.municipality B		1.82 (1.8 to 1.84)*	1.84 (1.82 to 1.87)*	1.86 (1.84 to 1.89)*
Nodefactor.municipality C		2.92 (2.87 to 2.97)*	2.89 (2.84 to 2.94)*	3.15 (3.1 to 3.2)*
Nodefactor.municipality D		3.98 (3.85 to 4.11)*	3.76 (3.64 to 3.88)*	3.95 (3.82 to 4.08)*
Nodefactor.municipality E		3.07 (2.94 to 3.21)*	2.86 (2.74 to 3)*	3.68 (3.52 to 3.85)*
Nodefactor.municipality G		1.65 (1.56 to 1.76)*	1.56 (1.47 to 1.66)*	1.58 (1.49 to 1.68)*
Nodematch.municipality		11.15 (10.95 to 11.36)*	11.36 (11.15 to 11.58)*	12.50 (12.26 to 12.74)*
Nodefactor. occupation.physicians (ref.)				
Nodefactor.occupation.nurses			0.71 (0.7 to 0.72)*	0.70 (0.69 to 0.7)*
Nodefactor.occupation.others			0.59 (0.58 to 0.6)*	0.83 (0.81 to 0.84)*
Nodematch.occupation			1.07 (1.05 to 1.08)*	1.06 (1.05 to 1.08)*
Nodefactor.service type.outpatient healthcare services (ref.)				
Nodefactor.service type.mental health				1.64 (1.61 to 1.67)*
Nodefactor.service type.others				0.46 (0.45 to 0.46)*
Nodematch.service type				1.17 (1.15 to 1.19)*
Bayesian information criterion	891 500.66	807 686.73	800 620.78	762 893.10

Nodefactor = number of times that nodes with a given level of a categorical nodal attribute appear within the edgeset (main effects).

Nodematch = number of edges whose incident nodes match on value of nodal attribute (homophily terms).

* p < 0.001.

All homophily terms (nodematches) were statistically significant (p values <0.001). Two professionals were more likely to share patient(s) if they had a similar occupation and, naturally, when they worked in the same municipality or within the same service type. The main effects (nodefactors) were also significant and suggested that being a physician predicts having more connections than belonging to other occupational groups. Municipality main effects suggested differences in patient-sharing between municipalities. The main effect of the service type was significant. The odds of sharing patients were 1.64 times higher among professionals who mainly provided mental health and substance use services compared with professionals (GPs and nurses) providing mainly outpatient healthcare services.

The ERGM analyses were based on professionals sharing patient(s) within 1 year time-period. Thus, in practice, the time-period when the professionals met the same patient could have been anything between 1 day and 1 year. It is possible, that the results could be different if a shorter time-period than 1 year was used. To test this, we ran an additional ERGM analysis using a maximum of 3 month time-period in patient-sharing relationships. This was done by defining the data in a way that it considered patient-sharing contacts that had occurred within a 3-month period starting from the first appointment a patient had within the study year. The results, presented in online supplemental file 2, are identical when the shorter time-period was used.

## Discussion

In this study, we examined the determinants of patient-sharing relationships between different professionals taking care of patients with mental health and substance use problems in the Finnish primary care. Our aim was to find out whether some attributes linked to the professionals in the healthcare register might explain network formation. Our study design exceeded many previous studies that have explored only networks between physicians and have thus ignored the multidisciplinary nature of real-world healthcare settings (for a review, see ref. 13).

The main results of the ERGMs suggest, first, that shared patients between professionals were more probable when they belonged to the same occupational group, provided similar types of services or worked in the same municipality. These results are in line with the homophily principle (the tendency of people to have contacts with similar others) which has received support in many studies in the healthcare context.^18 27 28^ Homophilous relationships may be useful for the professionals when they enhance, for example, active information or advice sharing among peers. Often, however, homophilous relationships (compared with heterophilous relationships between different professionals) are dreaded to hinder multidisciplinary teamwork and are thus undesirable from the perspective of healthcare organisations.^18 29^

Second, our results suggested that being a physician predicted having more connections than belonging to other occupational groups. This result seems to emphasise the crucial role of physicians in primary care (and perhaps especially among patients with mental health or substance use related service needs). Physicians are the only occupation group that is allowed to make diagnoses and decisions on medication for a patient, and thus the other professionals are required to collaborate (especially) with them. Also, physicians themselves need contacts to other professionals to make referrals or consult on issues that are on the field of other professionals’ expertise.

Third, we found that shared patients among different professionals were more probable when the patients were shared with the professionals working within mental health or substance use services. In the healthcare register that we used, these service types are used with patients that need psychiatric outpatient services or substance use related services. Also, the professionals offering these services and using these service codes are supposed to be experts in this field, for example, psychiatric nurses. The result that there were shared patients across municipalities, occupational groups and different services within patients using mental health services is promising if it indicates that taking care of mental health issues is integrated with primary care services as is desired.^2^ Based on our results, it appears that at least the patients with most severe needs in mental health or substance use services are being treated with multidisciplinary teams.

The results may in practice reflect the functioning of the Finnish health system, which promotes to improve the collaboration between different professionals. This is highlighted especially in the results which showed that physicians and mental health professionals were the most common parties in patient-sharing networks of the studied patient group. Future studies in different healthcare systems could benefit from identifying the necessary actors and the optimal combination of collaboration between them as an important predecessor for developing and evaluating highly integrated services.

Our twofold results (that relationships were mainly homogenous but also probable across some of the services and occupation groups) raise a question, what kind of professional network would be most suitable for patients with mental health or substance use issues. Lorant and colleagues^30^ have addressed this issue and found that it depends on what is the goal of the network. In their study, when continuity of care was the priority, the networks were found to be homophilous, but when social integration was the priority, the networks were heterophilous.^30^ Some of the previous patient-sharing network studies have found other kinds of outcomes, such as treatment costs, to be related with certain network structures.^31 32^ Thus, there is no single optimal network structure, as the most appropriate network is dependent on the desired outcomes of the network.^30^ Identifying the relevant network measures and their relationships to different network outcomes by using a multi-level approach in the healthcare context is an important aspect in future research.^33^

### Strengths and limitations

Our study serves as the first ambitious attempt to explore SNA using the large Finnish healthcare register which includes all the different professionals working in public and private sectors in primary care. However, one limitation was that we used data that included information only on a limited area in Finland and visits only from a 1 year time-period. It is possible that the results might have been changed, if other areas in Finland were explored or had the time-period included several years or some other year. However, we had to limit the number of visits to a somewhat controllable level to have the needed capacity for running the analyses using R and exploring the services in a certain geographical area seemed suitable for this purpose.

Another challenge was how to reliably identify the patients who had visited primary care services for reasons relating to mental health or substance use. The service type codes in the register generally represent more on which services the professionals operate under, rather than describe the nature of the visit (ie, the needs of the patient). For example, from the outpatient healthcare services (GP and nurse visits), it is almost impossible to separate the reason for the visit, because the professionals are instructed to use the same code for all kinds of visits within the service type. Insufficient information regarding mental health services at the primary care level in Finnish health registers has been noticed also elsewhere and actions to improve the content of the registers have been taken.^10^

Following many previous studies using administrative data, we assumed that sharing same patients generally indicates that the professionals also work together and share work-related information with each other. However, this interpretation cannot be confirmed by using the register data. A reason for a patient to visit several different professionals might be due to the complex needs of the patient or the referral policies within the organisations. Other possible explanations might be changes within service structures (due to COVID-19 pandemic or other reasons) or employee turnover.

An important methodological strength in our study was the use of ERGMs which account for the dependency of network structures and have been used previously in few studies (see ref. 13). However, this method is by no means the only method, that can be used when examining patient-sharing relationships. Many other SNA measures, such as different centrality measures, have had a statistically significant impact on outcomes such as quality of care.^34^ Future studies should further explore the use of different measures and aim at finding out the most promising and suitable measures for different healthcare contexts and outcomes.

## Conclusion

We were able to find some patterns (professional groups and service types) that describe patient-sharing networks in primary care beyond the municipality level. Exploring further administrative data, such as healthcare registers, might offer interesting and useful insights for understanding the patient-sharing relationships better in healthcare. Emphasis should however be put on developing the registers for administrative, implementation and research purposes and designing studies that combine and use the register data alongside information collected from other relevant source(s).

## supplementary material

10.1136/bmjopen-2024-089111 online supplemental file 1

10.1136/bmjopen-2024-089111 online supplemental file 2

## Data Availability

Data may be obtained from a third party and are not publicly available.
