# Supplementary material for: Patient-sharing networks among Finnish primary healthcare professionals taking care of patients with mental health or substance use problems: a register study
Source: BMJ Open. 2025 Jan 2;15(1):e089111. doi: 10.1136/bmjopen-2024-089111 (PMC11749436; doi:10.1136/bmjopen-2024-089111)
Supplement: online supplemental file 1 [file bmjopen-15-1-s001.pdf]

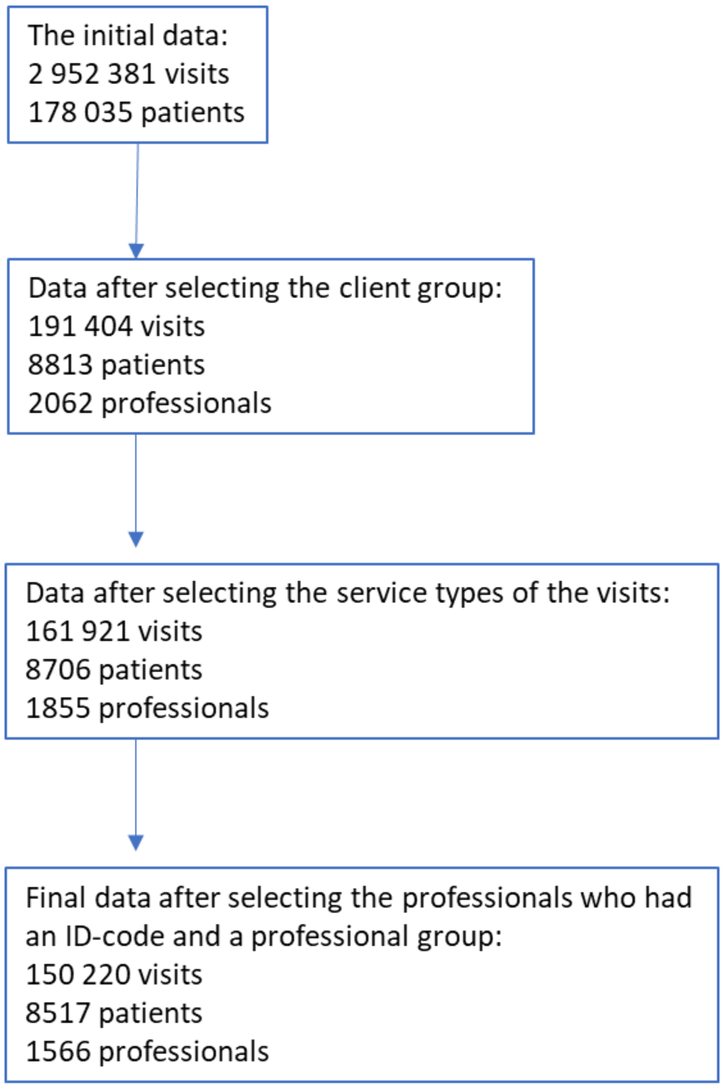

Supplement 1_online_supp. The number of visits, patients and professionals in different phases of constructing the register data.
