# Supplementary material for: Patient-sharing networks among Finnish primary healthcare professionals taking care of patients with mental health or substance use problems: a register study
Source: BMJ Open. 2025 Jan 2;15(1):e089111. doi: 10.1136/bmjopen-2024-089111 (PMC11749436; doi:10.1136/bmjopen-2024-089111)
Supplement: online supplemental file 2 [file bmjopen-15-1-s002.pdf]

Supplement 2_online_supp. Table. ERGM analysis of municipality, occupational group, and service type (using time frame 3 months).

	OR (CI)			
	**Model 1**	**Model 2**	**Model 3**	**Model 4**
Density (edges)	0.13 (0.13-0.13)***	0.02 (0.02-0.02)***	0.03 (0.02-0.03)***	0.03 (0.03-0.03)***
Nodefactor.municipality F (ref.)				
Nodefactor.municipality A		2.45 (2.39-2.52)***	2.54 (2.48-2.61)***	2.86 (2.79-2.93)***
Nodefactor.municipality B		1.92 (1.91-1.96)***	1.96 (1.94-1.99)***	1.99 (1.97-2.02)***
Nodefactor.municipality C		2.95 (2.9-3)	2.93 (2.88-2.98)***	3.19 (3.13-3.24)***
Nodefactor.municipality D		3.83 (3.7-3.97)***	3.62 (3.5-3.75)***	3.94 (3.8-4.09)***
Nodefactor.municipality E		3.36 (3.21-3.52)***	3.12 (2.97-3.27)***	3.93 (3.74-4.12)***
Nodefactor.municipality G		1.76 (1.65-1.87)***	1.66 (1.56-1.77)***	1.69 (1.59-1.8)***
Nodematch.municipality		12.16 (11.93-12.4)***	12.39 (12.15-12.63)***	13.58 (13.32-13.85)***
Nodefactor. occupation.physicians (ref.)				
Nodefactor.occupation.nurses			0.72 (0.71-0.73)***	0.70 (0.7-0.71)***
Nodefactor.occupation.others			0.60 (0.59-0.61)***	0.83 (0.82-0.84)***
Nodematch.occupation			1.06 (1.04-1.07)***	1.05 (1.04-1.07)***
Nodefactor.service type.outpatient health care services (ref.)				
Nodefactor.service type.mental health				1.63 (1.6-1.66)***
Nodefactor.service type.others				0.46 (0.46-0.47)***
Nodematch.service type				1.17 (1.15-1.19)***
BIC	838972.66	755929.07	749596.46	715648.24

***$p < 0.001$

Nodefactor = number of times that nodes with a given level of a categorical nodal attribute appear within the edgeset (main effects)

Nodematch = number of edges whose incident nodes match on value of nodal attribute (homophily terms)
