# Supplementary material for: Patient-sharing networks among Finnish primary healthcare professionals taking care of patients with mental health or substance use problems: a register study
Source: BMJ Open. 2025 Jan 2;15(1):e089111. doi: 10.1136/bmjopen-2024-089111 (PMC11749436; doi:10.1136/bmjopen-2024-089111)
Supplement: Supplementary file 3 [file bmjopen-2024-089111.reviewer_comments.pdf]

PEER REVIEW HISTORY

BMJ Open publishes all reviews undertaken for accepted manuscripts. Reviewers are asked to complete a checklist review form and are provided with free text boxes to elaborate on their assessment. These free text comments are reproduced below.

ARTICLE DETAILS

Title (Provisional)

Patient-sharing networks among Finnish primary health care professionals taking care of patients with mental health or substance use problems: A register study

Authors

Hietapakka, Laura; Sinervo, Timo; Väisänen, Visa; Niemi, Ripsa; Gutvilig, Mai; Linnaranta, Outi; Suvisaari, Jaana; Hakulinen, Christian; Elovainio, Marko

---

VERSION 1 - REVIEW

**Reviewer**	**1**
**Name**	**Romero-Sandoval, Natalia**
**Affiliation**	**Universidad Internacional del Ecuador, Quito, School of Medicine**
**Date**	**15-Aug-2024**
**COI**	**I have no conflicts of interest to disclose.**

General comments

This is a particularly important study for the analysis of patient-sharing networks among primary health care professionals taking care of patients with mental health or substance use problems

I'd like to highlight specific comments.

Specific comments

Abstract section

Lines 20: This sentence ("...use a sample...") introduces confusion into the abstract and strays somewhat from the study design, so I would like to suggest that it be clarified.

Conclusion: The first statement looks a little bit confusing. I'd like to recommend not using the terms "homophily hypothesis". The conclusion is clear using the statement into the parenthesis.

Introduction section

Page 6, Line 41-46: Although the authors present two characteristics of the health service system, I suggest that they include a brief description of the health system, e.g. organization and governance, financing, service delivery, coordination between levels of care, is the primary care level the gateway to specialized services at the secondary and tertiary levels of care, referral process between levels. Thus, the paragraph between lines 57 on page 6 and line 16 on page 7 should be restructured.

Results section

Page 15, Lines 47-50: This principle has not been described before. It is suggested to include an explanation in the Methodology section. On the other hand, a brief definition is presented in the Discussion section, page 17, lines 50-52. It is suggested to order the presentation of the definition of this characteristic.

Discussion section

For the sentence described between lines 36 to 41, page 17, include the references of the studies to which the authors refer.

The main findings should also be discussed under the structure and functioning of the Finnish health system (e.g. coordination between levels of care). To my knowledge, the Finnish health system is one of the best in the world and the results of this analysis could be very useful for health systems that are based on integrated networks of health services.

**Reviewer**	**2**
**Name**	**Uddin, Shahadat**
**Affiliation**	**The University of Sydney, Centre for Complex Systems Research**
**Date**	**25-Sep-2024**
**COI**	**I confirm that I don't have any conflict of interest with this submission.**

This is an interesting article that addresses an important research issue using some sophisticated methods (e.g., ERGM).

1. Please include details about the construction process from bipartite to unipartite networks (https://doi.org/10.1038/srep20222). The authors could consider network attributes from different levels.

2. It would be good to include some details of the ERGM (https://doi.org/10.1002/sim.5770).

3. In addition to considering a shared node (A patient-sharing relationship was defined between two professionals if the same patient had visited both of them at least once), the authors could consider a timeframe. The difference could be one day or one year. There must be a difference between such a large difference gap.

4. please consider some of the other SNA methodologies, in addition to ERGM, for analysing such bipartite networks (10.1109/ASONAM.2014.6921647); e.g., how they evolve and does such evolution have any impact on the target measure, etc.

VERSION 1 - AUTHOR RESPONSE

Reviewer: 1

Comment 1: This is a particularly important study for the analysis of patient-sharing networks among primary health care professionals taking care of patients with mental health or substance use problems. I'd like to highlight specific comments.

Our response: We thank the reviewer for the positive feedback.

Comment 2: Abstract section: Lines 20: This sentence ("...use a sample...") introduces confusion into the abstract and strays somewhat from the study design, so I would like to suggest that it be clarified.

Our response: We have now modified the sentence in Abstract section to clarify it. In the text: "We used the register data covering the visits for the service providers of seven municipalities, adult patients with at least one visit to a health and social service center (HSSC) within one of the municipalities, and visits during year 2021."

Comment 3: Conclusion: The first statement looks a little bit confusing. I'd like to recommend not using the terms "homophily hypothesis". The conclusion is clear using the statement into the parenthesis.

Our response: We thank the reviewer for pointing that out. We removed the term "homophily hypothesis" and the first sentence of the Conclusions is now in the text: "Patient-sharing contacts were mainly homogenous supporting the tendency of people to have connections with similar others."

Comment 4: Introduction section: Page 6, Line 41-46: Although the authors present two characteristics of the health service system, I suggest that they include a brief description of the health system, e.g. organization and governance, financing, service delivery, coordination between levels of care, is the primary care level the gateway to specialized services at the secondary and tertiary levels of care, referral process between levels. Thus, the paragraph between lines 57 on page 6 and line 16 on page 7 should be restructured.

Our response: We have now added information on the Finnish health system to the two paragraphs in the introduction. In the text: "Second, in Finland health services are separated under different administrative sectors and funding sources. During the data collection of this study, the Finnish health services were organized by the municipalities (local authorities) that were responsible for financing primary and secondary care services. Most of the health spending in Finland goes to outpatient care [8]. Outpatient health services, including mental health services, are provided by health and social service centers (HSSCs) and outpatient departments of psychiatric hospitals [9]. Other comparable service providers are the Finnish Student Health Service and occupational health care. These primary care level

services are the first-contact care and act as gatekeepers to secondary care. Psychiatric services in secondary care are responsible for treating the more severe mental disorders but a referral is needed to access these services [8,9].
Third, mental health services have been integrated to primary health care with different intensities [2]. In Finland, primary health care professionals (physicians and nurses) either guide the patient to contact the mental health professionals or consult them on their own. The role of psychiatric nurses has been increased and psychosocial treatments and psychotherapies can be started in HSSCs [11]. In some municipalities or organizations, the mental health professionals sometimes work under the same administration or even in the same team as other primary care professionals in HSSCs, and in other cases they might work in separate organizations but can be consulted when needed. These local variations of how the mental health services have been arranged can either enhance or hinder collaboration between professionals and organizations."

Comment 5: Results section: Page 15, Lines 47-50: This principle has not been described before. It is suggested to include an explanation in the Methodology section. On the other hand, a brief definition is presented in the Discussion section, page 17, lines 50-52. It is suggested to order the presentation of the definition of this characteristic.

Our response: The explanation of the homophily principle was indeed not in the right place considering the order of its presentation in the text. We changed the text so, that the explanation comes first in the Methods section and in line with this did a few small modifications in other parts of the text. In the text (Methods section): "We used two ERGM terms, called nodefactor and nodematch, to explain the structures of the patient-sharing networks. Nodefactor refers to the number of times nodes with a certain categorical nodal attribute appear within the edgeset. This was used for testing for example whether being a certain professional predicted having more connections than belonging to other occupational groups. Nodematch refers to the number of edges whose incident nodes match on value of a certain nodal attribute. This is related to the so-called homophily principle, i.e. the tendency of people having contacts with similar others [18]. We used nodematch to test, whether the professionals had more contacts with professionals working in the same municipality, same profession or within same service type as they did."

The modified sentence in the Results section: "All homophily terms (nodematches) were statistically significant (p-values <.01)."

The modified sentence in the Discussion section: "These results are in line with the homophily principle (the tendency of people to have contacts with similar others) which has received support in many studies in health care context [24–26]".

Comment 6: Discussion section: For the sentence described between lines 36 to 41, page 17, include the references of the studies to which the authors refer.

Our response: We have now added a review article to that sentence, in the text: "Our study design exceeded many previous studies that have explored only networks between physicians and have thus ignored the multidisciplinary nature of real-world health care settings (for a review, see [13])."

Comment 7: The main findings should also be discussed under the structure and functioning of the Finnish health system (e.g. coordination between levels of care). To my knowledge, the Finnish health system is one of the best in the world and the results of this analysis could be very useful for health systems that are based on integrated networks of health services.

Our response: We thank the reviewer for the compliment regarding the Finnish health system. We have now added a paragraph to the Discussion part reflecting the results under the Finnish health system. In the text: "The results may in practice reflect the functioning of the Finnish health system, which promotes to improve the collaboration between different professionals. This is highlighted especially in the results which showed that physicians and mental health professionals were the most common parties in patient-sharing networks of the studied patient group. Future studies in different healthcare systems

could benefit from identifying the necessary actors and the optimal combination of collaboration between them as an important predecessor for developing and evaluating highly integrated services.”

Reviewer: 2

Comment 1: This is an interesting article that addresses an important research issue using some sophisticated methods (e.g., ERGM).

Our response: We thank the reviewer for the positive feedback.

Comment 2: Please include details about the construction process from bipartite to unipartite networks (https://doi.org/10.1038/srep20222). The authors could consider network attributes from different levels.

Our response: Considering network attributes from different levels is a good point. In this study, our initial data had an underlying community structure, because the data represented visits from several municipalities, and thus most of the patients as well as professionals were involved in only one municipality. We also were not able to compare possible network measures in relation to any service level outcomes. However, this is important when planning future studies and we have added some text on this to the discussion part. In the text: “Identifying the relevant network measures and their relationships to different network outcomes by using a multi-level approach for network studies in the health care context is an important aspect in future research [33].

Comment 3: It would be good to include some details of the ERGM (https://doi.org/10.1002/sim.5770).

Our response: This is a very good suggestion. We have now added some details to the Statistical analyses part. In the text: “One important advantage of ERGMs is, that as opposite to regression analyses, they can be used on a relational data (which patient-sharing data always is) since they do not require assumptions of independence. ERGMs are also scalable as the structure of the graph is represented by locally determined explanatory variables and the choice of these variables is quite flexible [17].”

Comment 4. In addition to considering a shared node (A patient-sharing relationship was defined between two professionals if the same patient had visited both of them at least once), the authors could consider a timeframe. The difference could be one day or one year. There must be a difference between such a large difference gap.

Our response: This is indeed an important comment. The timeframe of a patient seeing different professionals can vary between one day and a year depending for example on the situation in the centre at the time. To check the results on a shorter time-period than whole year, we did additional analyses in which we defined the time limit for patient-sharing to be at maximum three months. The results were very similar to the original results, suggesting that in our data there might not be that huge differences on patient-sharing relationships based on different timeframes. We present the additional analyses in the text in Results section and in the new supplement table 2. In the text: “The ERGM analyses were based on professionals sharing patient(s) within one year time-period. Thus, in practice the time-period when the professionals met the same patient could have been anything between one day and one year. It is possible, that the results could be different if a shorter time-period than one year was used. To test this, we ran an additional ERGM analysis using a maximum of three-month time-period in patient-sharing relationships. This was done by defining the data in a way that it considered patient-sharing contacts that had occurred within a three-month period starting from the first appointment a patient had within the study year. The results, presented in Supplement 2, are identical when the shorter time-period was used.”

Comment 5: please consider some of the other SNA methodologies, in addition to ERGM, for analysing such bipartite networks (10.1109/ASONAM.2014.6921647); e.g., how they evolve and does such evolution have any impact on the target measure, etc.

Our response: We thank the reviewer for this comment, we indeed had not discussed the possible relevance of other SNA methodologies in the manuscript. We added now a paragraph to the Strengths and limitations part to point out, that other measures also exist and can be useful in future studies. In the text: "An important methodological strength in our study was the use of ERGMs which account for the dependency of network structures and have been used previously in few studies (see [13]). However, this method is by no means the only method, that can be used when examining patient-sharing relationships. Many other SNA measures, such as different centrality measures have had a statistically significant impact on outcomes such as quality of care [34]. Future studies should further explore the use of different measures and aim at finding out the most promising and suitable measures for different healthcare contexts and outcomes."

VERSION 2 - REVIEW

**Reviewer**	**2**
**Name**	**Uddin, Shahadat**
**Affiliation**	**The University of Sydney, Centre for Complex Systems Research**
**Date**	**23-Nov-2024**
**COI**	**I confirm that I don't have any conflict of interest with this submission.**

There are a few instances of missing spaces after the full-stop character. Please correct them.
